# College Commencements

**Published:** 1893-06

**Authors:** 


BUFFALO MEDICAL AND SURGICAL JOURNAL
A MONTHLY REVIEW OF MEDICINE AND SURGERY.

*EDITORS:*
THOMAS LOTHROP, M. D. - - WM. WARREN POTTER, M. D.
All communications, whether of a literary or business character, should be addressed to the managing editor: 284 FRANKLIN STREET, BUFFALO, N. Y.

VOL. XXXII. JUNE, 1893. No. 11.

### COLLEGE COMMENCEMENTS.

### MEDICAL DEPARTMENT OF NIAGARA UNIVERSITY.

THE tenth annual commencement of this school occurred Thursday, May 4, 1893, and in every particular, with the possible exception of the weather, was a perfect success.

The Alumni Association held its eighth annual meeting in the morning at ten o'clock in the amphitheatre of the college building, on Ellicott street. Professor Alvin A. Hubbell, in his address of welcome, reviewed briefly the history of the institution from its beginning in 1883: "It was at first domiciled in the Buffalo Hospital of the Sisters of Charity, with a dissecting-room, anything but attractive and comfortable, in an adjoining building. These quarters were, however, soon found to be inadequate and part of the work was taken to the rooms in the Y. M. C. A. Building, on Mohawk street. Later, rooms for dissecting were used in the old Court House building, corner of Clinton and Ellicott streets.

"In 1884-5, the present premises were purchased and part of the present building was erected. This was ready for occupancy at about holiday time of that year, and the didactic work of the school was then transferred from the Y. M. C. A. Building, the chemical work from Dr. Davidson's private laboratory, and the dissection from the old Court House building. The opening of the new building was an important event to the school, and both teachers and students set themselves to their tasks with a new inspiration. For some time the conveniences of the building sufficed, but as new demands were created and new needs were to be met, it was decided to enlarge the building. This was done in 1890, when the capacity was more than doubled, giving the present commodious and well-arranged quarters, which equal, at least in convenience and adaptation to the requirements of medical teaching, if not in expensive and decorative architecture, that of any college building in the country."

Of the changes in the faculty, Dr. Hubbell spoke as follows:

"The faculty of today is not, however, the faculty of 1883-4. The professorial body was then made up of eleven physicians and one lawyer. It would be more difficult, it seems to me, to select from the rank and file of the professions a more capable body of men for teaching purposes. Dr. Gay and Dr. Davidson early passed over to the "great majority." Dr. Tremaine was prevented by ill-health from doing the work developing upon his position, and he retired from the activities of the school; but it is still honored by his name and prestige as Emeritus Professor of the Principles and Practice of Surgery and Clinical Surgery. Dr. Stockton was invited to other fields of labor. Dr. Heath and Dr. Daniels separated from the faculty later. All of these men were excellent teachers, and by their death or withdrawal the chairs they occupied suffered severe loss. Mr. Congdon, who was so efficient in the organization of the school, was not able to attend to the duties of the position to which he was appointed and soon gave place to another.

"Now the institution has in its possession the resignation, to take effect at the close of this year, of Dr. Fell, who has most devotedly and self-sacrificingly applied himself to the department of physiology and microscopy.

"Of the original twelve, therefore, we have left only today Drs. Cronyn, Lothrop, Ingraham, and your humble servant.

"Among the later accessions to the faculty, Drs. Campbell, S. T. Clark, and F. H. Potter have gone to their silent rest. Drs. Wheeler, Edward Clark, and Persons have sought other fields."

Dr. Clarence B. Le Van, President of the Alumni Association, in his annual address, reviewed from the standpoint of a student, the history and growth of the school, concluding his remarks by criticising the county medical societies, particularly that of Erie county.

At the close of Dr. Le Van's remarks, the election of officers for the ensuing year took place, with the following result: Dr. John M. Hewitt, President; Dr. A. W. Bayliss, First Vice-President; Dr. E. M. Dooley, Second Vice-President; Dr. W. H. Norrish, Secretary; Executive Committee, Drs. J. M. Meyers, L. A. Hanley, and J. Henry Dowd.

Resolutions in relation to Dr. William R. Griffis, of the Alumni

Association, who died during the year, were adopted. Among the older graduates in attendance were Dr. Joseph J. Kane, of Washington, and Dr. Sweeney, of New Orleans.

In the afternoon the Alumni Association met in the amphitheatre, when papers were read and discussed. The readers were Dr. J. Henry Dowd, of Buffalo; Dr. Edward Torrey, of Allegany, N. Y., and Dr. L. J. McAdam, of Buffalo.

Dr. Dowd's paper will be found on page 660 of the Journal.

The graduating exercises occurred at the Star Theatre, at 8.15 P. M. The pretty theatre was crowded to overflowing. The Right Rev. Stephen V. Ryan, Chancellor of the University, the Rev. Thomas Slicer, the Faculty of the Medical Department of the University, and the Board of Medical Examiners occupied chairs on the stage, while the graduating class occupied the front tier of chairs in the orchestra circle. Dr. Thomas Lothrop, Vice-President of the Faculty, presided over the exercises, and after a brief congratulatory address by Bishop Ryan, delivered the decennial address. (The address is published in full in this number of the Journal.)

The following doctors then received their degrees: Herbert William Fudge, Elmira; William Gould Taylor, Buffalo; Daniel Francis White, Saratoga Springs; Frederick Michael Boyle, Auburn; Patrick Henry Hourigan, Oswego; Henry Jefferson Newton, Fredonia; Thomas Francis Healy, Birmingham Conn.; George Heslop Grey, Glanford, Ont.; James Allen Blair, Arnot, Pa.; James Henry Meehan, Niagara Falls. Earl Lothrop received the degree as proxy for George Heslop Grey. Mr. Grey was taken suddenly sick and was unable to be present. The Rev. Mr. Slicer closed the exercises with a most excellent address to the graduates. Perhaps no more able and impressive remarks were ever made to a body of graduates in Buffalo, and the Journal regrets very much at not being able to reproduce them.

The banquet at the Tifft was a fitting finale to the day's exercises; the party, numbering about sixty persons, included the officers of the University, professors, alumni, and invited guests. Clarence B. Le Van, M. D., acted as toastmaster. The following toasts were responded to: "The University," by the Rev. P. C. Kavanaugh; "The Medical Department," by Eugene A. Smith, M. D.; "The Clergy," by the Rev. Dr. Aaron; "The Board of Health," by Ernest Wende, M. D.; "Dentistry," Dr. Frank Low; "The Legal

Profession," George E. Bird; and "The Class of '93," Dr. W. G. Taylor.

The party separated at an early hour in the morning, convinced that this commencement marks an important epoch in the history of the institution, and every one satisfied, yea, gratified with the successful termination of the day's exercises.

---

MEDICAL DEPARTMENT—UNIVERSITY OF BUFFALO.

The commencement exercises of this institution partook of more than the ordinary amount of interest and importance this year by virtue of the dedication of the new college building on High street. Tuesday, May 2, 1893, was the day appointed for these exercises, and favored with pleasant weather. A large number of out-of-town alumni, together with resident alumni, assembled in alumni hall at 10.30 A. M. In the absence of Dr. G. R. Hopkins, of Silver Creek, president of the Alumni Association, Dr. W. C. Phelps, of Buffalo, presided. In his address of welcome, Professor Roswell Park referred to the completion of the new building, its beauty, usefulness, and adaptation to every need, declaring it to be the most hospitable, comfortable, serviceable, and attractive to be found among the medical schools in the world. His hearers seemed to share the same belief.

The following were elected officers of the Alumni Association for 1893-4: President, W. C. Phelps, Buffalo; First Vice-President, Ernest Wende, Buffalo; Second Vice-President, E. H. Woolsey, Oakland, Cal.; Third Vice-President, P. W. Van Peyma, Buffalo; Fourth Vice-President, Mrs. Jane W. Carroll, Buffalo; Fifth Vice-President, D. A. Currie, Englewood, N. J.; Permanent Secretary, J. J. Walsh, Buffalo; Recording Secretary, F. B. Willard, Buffalo; Treasurer, H. U. Williams; Trustees: Henry Lapp, Clarence; F. E. L. Brecht, E. C. W. O'Brien, Joseph Fowler, Mahlon B. Folwell, Buffalo.

Executive Committee: W. H. Bergtold, W. C. Phelps, John Parmenter, Secretary Medical Faculty, F. P. Vandenberg, and H. H. Bingham.

The Chairman was instructed to appoint a committee of five to consider and report on the advisability of establishing a school of veterinary medicine in connection with the University.

The afternoon session was largely attended. Papers were read by Dr. Paul F. Mundé, of New York; Dr. C. C. Frederick, of Buffalo, and Dr. F. Stanbro, of Springville, N. Y.

The commencement exercises occurred at Music Hall at 8.30 P. M. Seated upon the stage were Chancellor E. C. Sprague, the officers of the University, Rev. Dr. Thos. Slicer, the faculties of the departments of medicine, pharmacy, and dentistry, friends and alumni of the University.

The Department of Medicine graduated forty-nine members.

The Chancellor announced that the Council of the University had conferred on Dr. Evan O'Neil Kane, of Kane, Pa., the honorary degree for distinguished contributions to medicine.

The honor roll was read as follows: 1, Albert E. Woehnert; 2, Burnell R. Johnson; 3, Albert T. Lytle; 4, William J. Squire; 5, Franklin W. Barrows; 6, Evangeline Carroll; 7, John Riordon; 8, Fred James Mann; 9, Fred H. Bailey; 10, Grant A. Neal.

Dr. Albert T. Lytle received honorable mention for a thesis on Plica Polonica.

The department of pharmacy granted diplomas to twenty, while the department of dentistry granted diplomas to five young men. This is the first class to graduate in dentistry, and if they live up to the oath administered by the Dean, this school need not fear that its alumni will degrade themselves or their alma mater.

Dr. Paul F. Mundè, of New York, then addressed the graduates, taking for his subject, Specialism in Medicine. Contrary to the generally accepted notion that the specialist is the creature of modern times, Prof. Mundè asserted that specialists have existed from the earliest times. He instanced such specialists as Laennec and Andral in diseases of the lungs; Siebold, Mariceau, and Smellie in obstetrics and gynecology; Bright and Rayer for diseases of the kidney; Cruveilhier, in pathological anatomy; Hunter, Bichat, Magendie, and Muller, for anatomy and physiology; Sir Astley Cooper in surgery, and Mesdames Boivin and Lachapelle in obstetrics, also came in for notice.

The speaker concluded his address with the following advice: "Do not begin by being a specialist. Start as a general practitioner. Become thoroughly trained as a physician, and then when you have grown in experience and have gained sufficient insight into the various branches of medicine to be able to judge for which special line your capacities and talents fit you, and to which your inclinations draw you, then *caeteris paribus*, circumstances and means permitting, adopt that specialty, either by a gradual acquaintance with it in the course of general practice, or by special study for a sufficient length of time." He counseled his hearers against being one-sided or routinists, and whether as specialists or general practitioners, never to forget that their first duty was to their patients.

The proceedings closed with the benediction, pronounced by the Rev. Dr. Slicer.

The Alumni Association of the Medical Department banqueted at the Tifft House under the toastmastership of Dr. W. C. Phelps, president of the Association.

